# Early effects of duloxetine on emotion recognition in healthy volunteers

**DOI:** 10.1177/0269881115570085

**Published:** 2015-03-10

**Authors:** Susan Bamford, Ian Penton-Voak, Verity Pinkney, David S Baldwin, Marcus R Munafò, Matthew Garner

**Affiliations:** 1 School of Psychology, University of Southampton, Southampton, UK; 2 School of Experimental Psychology, University of Bristol, Bristol, UK; 3 Clinical and Experimental Sciences, Faculty of Medicine, University of Southampton, Southampton, UK; 4 UK Centre for Tobacco and Alcohol Studies, University of Bristol, Bristol, UK; 5 MRC Integrative Epidemiology Unit at the University of Bristol, Bristol, UK

**Keywords:** Depression, anxiety, duloxetine, emotion recognition, sadness

## Abstract

The serotonin-noradrenaline reuptake inhibitor (SNRI) duloxetine is an effective treatment for major depression and generalised anxiety disorder. Neuropsychological models of antidepressant drug action suggest therapeutic effects might be mediated by the early correction of maladaptive biases in emotion processing, including the recognition of emotional expressions. Sub-chronic administration of duloxetine (for two weeks) produces adaptive changes in neural circuitry implicated in emotion processing; however, its effects on emotional expression recognition are unknown. Forty healthy participants were randomised to receive either 14 days of duloxetine (60 mg/day, titrated from 30 mg after three days) or matched placebo (with sham titration) in a double-blind, between-groups, repeated-measures design. On day 0 and day 14 participants completed a computerised emotional expression recognition task that measured sensitivity to the six primary emotions. Thirty-eight participants (19 per group) completed their course of tablets and were included in the analysis. Results provide evidence that duloxetine, compared to placebo, may reduce the accurate recognition of sadness. Drug effects were driven by changes in participants’ ability to correctly detect subtle expressions of sadness, with greater change observed in the placebo relative to the duloxetine group. These effects occurred in the absence of changes in mood. Our preliminary findings require replication, but complement recent evidence that sadness recognition is a therapeutic target in major depression, and a mechanism through which SNRIs could resolve negative biases in emotion processing to achieve therapeutic effects.

## Introduction

Emotion recognition is a core feature of social interaction, and deficits in emotion recognition have been associated with a range of mental health problems, including schizophrenia (Addington et al., 2006), alcoholism (Philippot et al., 1999), autism (Celani et al., 1999), bipolar disorder (Derntl et al., 2009), depression (Rubinow and Post, 1992), and anxiety disorders (Button et al., 2013; Garner et al., 2009). A number of studies have also demonstrated that some antidepressant pharmacotherapies can modify the recognition of emotion, leading to the suggestion that antidepressants may exert some of their therapeutic effects via the early remediation of these cognitive biases prior to changes in clinical symptoms (Harmer and Cowen, 2013).

Early studies examined the effect of brain serotonin on emotion recognition. For example, reducing levels of serotonin in healthy volunteers through tryptophan depletion can reduce the recognition of fear (Harmer et al., 2003b), while tryptophan supplementation increases the recognition of happiness (Murphy et al., 2006). Acute increases in brain serotonin following the administration of a single (20 mg) dose of the selective serotonin reuptake inhibitor (SSRI) citalopram may increase recognition of fear (Browning et al., 2007) and happiness (Harmer et al., 2003), and modulate amygdala responses to emotional faces (Anderson et al., 2007; Del-Ben et al., 2005; Murphy et al., 2009).

Short-term (i.e. sub-chronic) administration of SSRIs can also modulate emotional expression recognition. For example, seven-day administration of citalopram increased the perception of happiness in ambiguous faces, and reduced the perception of negative fearful, angry and disgusted facial expressions (Harmer et al., 2004). Sub-chronic SSRI administration can also reduce amygdala response to negative faces following seven-day (Harmer et al., 2006), 10-day (Windischberger et al., 2010) and 21-day (Arce et al., 2008) administration. Although differences in emotion recognition following acute (i.e. single-dose) versus sub-chronic modulation of serotonin remain unclear (Pringle et al., 2013), these results together suggest that adaptive changes in emotion recognition (and activation in associated neural structures such as amygdala) could mediate the clinical response to SSRIs (Harmer and Cowen, 2013).

Drugs that target noradrenergic mechanisms are also widely used to treat mood and anxiety disorders (Nutt et al., 2006; Kalk et al., 2011); however, in contrast to SSRIs, comparatively little is known about their effects on emotion recognition. In healthy volunteers a single 80 mg dose of the beta-adrenoceptor blocker propranolol impaired the recognition of sad faces (Harmer et al., 2001), though this effect may in part result from possible serotonergic effects of propranolol at this high dose, given observations that propranolol has antagonist effects at presynaptic 5-HT autoreceptors (Audi et al., 1989). A single 4 mg dose of the selective noradrenaline reuptake inhibitor (NRI) reboxetine increased recognition of happiness (Harmer et al., 2003a).

Likewise a 60 mg single dose of the serotonin-noradrenaline reuptake inhibitor (SNRI) duloxetine increased recognition of happiness, and also increased the recognition of disgust, perhaps due to the poor tolerability of the 60 mg dose in this study (Harmer et al., 2008).

To date only one study has examined the effects of sub-chronic duloxetine administration on face processing. Following two-week administration of 60 mg duloxetine, healthy participants displayed reduced activity in the extended amygdala circuitry during an emotional face matching task in the absence of behavioural effects (van Marle et al., 2011). Imaging studies are typically not designed to reveal behavioural effects, as these can confound the interpretation of imaging data. Thus it remains unclear whether sub-chronic administration of duloxetine can modulate behavioural measures of emotion recognition. Consequently we compared the effects of 14-day administration of 60 mg duloxetine (titrated from 30 mg after day 3, in an attempt to improve tolerability) and placebo on emotion recognition in healthy volunteers.

## Materials and methods

### Participants

Forty healthy participants (20 females; mean age 24.7 years) were recruited from the local community and attended a pre-test screening session, during which they underwent a structured diagnostic interview based on Diagnostic and Statistical Manual of Mental Disorders, fourth edition (DSM-IV) criteria (Mini-International Neuropsychiatric Interview – MINI) (Sheehan et al., 1998). Exclusion criteria included recent use of medication (past eight weeks, except for topical treatment; occasional aspirin or paracetamol; oral, injectable or skin patch contraception), pregnancy, history of asthma/respiratory illness, high blood pressure (> 140 systolic and/or 90 diastolic), cardiovascular disease, migraines, current or lifetime history of psychiatric illness (including lifetime history/family history of panic attacks), regular smoking (more than six cigarettes/day), under- or over-weight (body mass index less than 18 or greater than 28 kg/m^2^), current or past drug or alcohol dependence and recent use of illicit drugs or alcohol (verified by breath test).

### Materials

**Figure 1.**
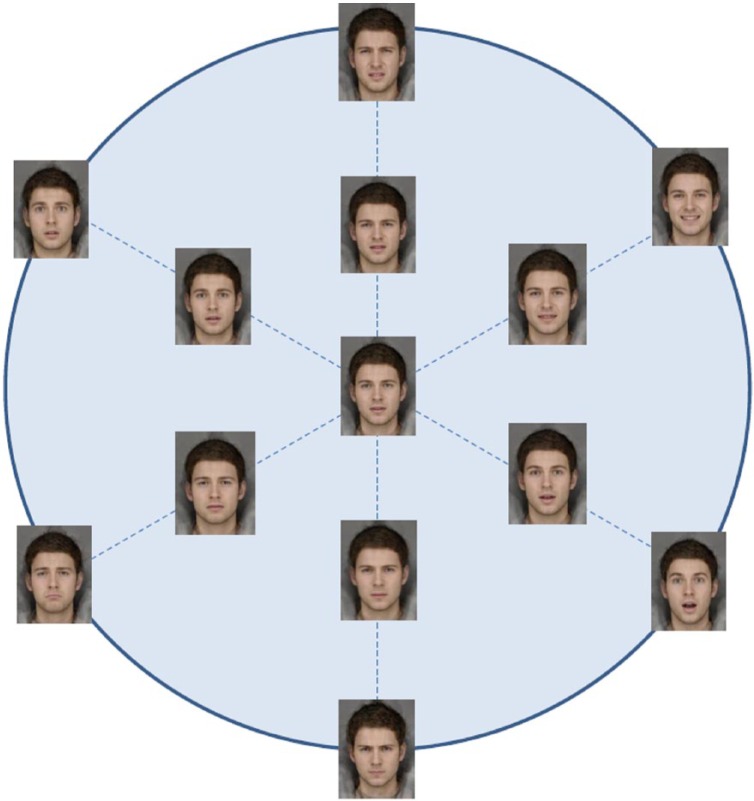
Emotional expression continua.

The facial expression stimuli for the emotion recognition task were created from photographs of 12 young adult male individuals photographed under controlled conditions. Each of these participants posed expressions (happy, sad, angry, disgusted, fearful, surprised and neutral) in a booth painted Munsell N5 grey, illuminated with 3 Verivide F20 T12/D65 daylight simulation bulbs in high-frequency fixtures (Verivide, UK), to reduce the effects of flicker. From the individual photographs, we constructed composite images of each of six emotions (happy, sad, angry, disgusted, fearful and surprised) using well-established methods (Tiddeman et al., 2001). In addition, we constructed a prototypical ‘emotional’ face constructed by compositing all 12 individuals in each of the six basic emotions plus neutral expressions. This face appears genuinely emotionally ambiguous, rather than neutral. We use this technique principally because of recent evidence which suggests that visual representations of emotion are better described as being coded with reference to a prototype of this sort, as opposed to a neutral face (Skinner and Benton, 2010). We created six 15-image morph sequences that ran along a linear continuum from this emotional prototype to each of the full-intensity emotions (90 stimuli in total) using standard techniques (Tiddeman et al., 2001) (Figure 1). So as to ensure that every face had some emotional signal, the first image in each sequence was a morph 5% along the dimension of ‘prototypical emotion’ to ‘full intensity’ of emotion.

### Procedure

Participants were randomised (stratified by sex) to receive either 14 days of duloxetine (60 mg per day, titrated from 30 mg after three days) or matched pill placebo (with sham titration). Group allocation was double blind, and participants were contacted by telephone on days 3, 6, 9 and 12 to confirm adherence and report any side effects or adverse events – none were reported. On day 0 (baseline) and day 14 participants attended the testing laboratory and completed self-report measures of anxiety (Generalised Anxiety Disorder Screener (GAD-7) with visual analogue scales ranging from 0 to 100, (Spitzer et al., 2006)), positive and negative affect (PANAS, Watson et al., 1988) blood pressure and heart rate (Omron M2 upper arm monitor), and a version of the Cambridge Cognition Emotion Recognition Task (http://www.camcog.com/emotion-recognition-task.asp) coded in E-Prime version 2 software. This is a six-alternative forced-choice paradigm which assesses sensitivity to each of the six primary emotions. In this task each trial began with a centrally displayed fixation cross, shown on-screen for between 1500 ms and 2500 ms. The 350 × 457 pixel face stimulus was presented for 150 ms, followed by a noise mask for 250 ms in order to prevent after-image effects (Cooper et al., 2008). Screen resolution was set to 1024 × 724 pixels. Participants were required to identify the emotion represented in each face as quickly and as accurately as possible, by using the mouse to click on the most appropriate descriptor from an array displayed on-screen (fearful, angry, happy, sad, disgusted and surprised). The descriptor array appeared on-screen for 10,000 ms, or until the participant responded. Each image was presented once, giving 90 trials in total. The images were viewed from a distance of approximately 70 cm, with image presentation being controlled using E-Prime software (PST Inc, Sharpsburg, PA, USA). Approximately 30 minutes after completing the Emotion Recognition Task on day 14, participants completed an experimental anxiety induction procedure (carbon dioxide challenge). This task required participants to complete an eye-tracking attention task both during the 20-minute inhalation of air enriched with 7.5% carbon dioxide, and the inhalation of normal air. Subjective anxiety and autonomic arousal were measured post-inhalation (protocol detailed in Garner et al., 2011; Garner et al., 2012 – data to be reported elsewhere). The study protocol was approved by the Ethics and Research Governance office at the University of Southampton. Participants received compensation (£100) for participating in the experiment.

### Statistical analysis

Our sample size of 40 provides in excess of 95% power at an alpha level of 5% to detect a large effect size (Cohen’s *d* = 0.85, based on the effect of a single dose of duloxetine on emotion recognition reported in Harmer et al., 2008). Mixed-model analyses of variance (ANOVAs) were used to explore the effects of drug (duloxetine, placebo), time (day 0, day 14), and drug × time on self-reported mood and anxiety, heart rate, and blood pressure. Accurate recognition of emotional expression is characterised by a high hit rate (ability to correctly identify an expression) and a low false-alarm rate (reduced tendency to mislabel a particular emotion). Following established methods (Wagner et al., 1993; Corwin, 1994) we calculated accuracy scores for each emotion and time-point that reflect the difference between hit and false-alarm rates, i.e. *p*(hits) minus *p*(false alarms). Positive scores (tending to 1) reflect greater accuracy. Distributions of accuracy performance scores varied substantially across emotions (F_Max_ variance ratio = 19.61 is above the threshold value of 3.32 that provides clear evidence of departures from homogeneity in samples of this size). Distributions were homogenous within each emotion, consequently we examined drug × time effects within each emotion separately. All analyses were conducted in IBM SPSS Statistics version 19. For completeness we report effect sizes (partial eta-squared), 95% confidence intervals and exact uncorrected *p* values. The conservative Bonferroni-corrected 5% alpha level conventionally used to evaluate statistical significance is 0.05/6 = 0.008; however, we direct readers to recent debate regarding the limitations of null-hypothesis significance testing (see Cumming, 2014).

## Results

### Characteristics of participants

Two participants (one duloxetine, one placebo) did not complete the course of tablets and were removed from the analysis (both participants reported forgetting to take two tablets). The final sample for analysis therefore consisted of 38 participants (50% female). The duloxetine and placebo groups did not differ in age or baseline levels of anxiety, mood or heart rate and blood pressure. In addition, there was no clear evidence for any effects of duloxetine on anxiety, mood, blood pressure or heart rate compared to placebo, with the exception of systolic blood pressure, which was slightly reduced on day 14 relative to day 0 in the placebo group only. Levels of anxiety (GAD-7) and PANAS remained within the normal range (normative means: PANAS positive = 31.3; PANAS negative = 16.0, Crawford and Henry, 2004; GAD-7 (scaled to 0–100 range) = 14.0, Löwe et al., 2008). A summary of participant characteristics by drug group and time is presented in Table 1.

**Table 1.** Participant characteristics in duloxetine and placebo groups on day 0 (baseline) and day 14 (post-drug).

	Duloxetine				Placebo				
	(*n* = 19)				(*n* = 19)				
Sex	9 male:10 female				10 male:9 female				
Age	24.5		(6.0)		24.9		(8.9)		
Body mass index	22.8		(2.3)		21.1		(1.6)		
	Day 0		Day 14		Day 0		Day 14		*p*
GAD-7	9.4	(6.2)	14.3	(8.9)	9.6	(5.1)	11.3	(6.3)	0.16
PANAS positive	34.9	(5.7)	33.6	(6.7)	35.3	(4.1)	33.6	(4.4)	0.83
PANAS negative	13.2	(2.9)	12.8	(2.4)	13.4	(2.5)	14.1	(3.6)	0.30
Heart rate	73.2	(10.9)	73.3	(8.5)	68.8	(11.2)	70.3	(10.9)	0.65
Systolic BP	124.8	(8.4)	124.3	(9.4)	125.1	(8.6)	119.2	(10.5)	0.06
Diastolic BP	71.6	(9.6)	71.2	(8.0)	69.5	(9.2)	66.2	(7.0)	0.27

Values represent mean (standard deviation). *P* value reflects drug × time interaction. GAD: Generalised Anxiety Disorder Screener; PANAS: positive and negative affect; BP: blood pressure.

The researchers responsible for participant testing (SB and VP) were unable to accurately determine drug-group membership (χ^2^ (1, *N* = 40) = 2.50, *p* = 0.113; correct allocations: placebo group = 13/20; duloxetine group = 12/20). There was some evidence that participants could identify their drug-group when asked on day 14, (χ^2^ (1, *N* = 40) = 10.4, *p* = 0.001); however, this was driven by those taking placebo (17/20 correct) rather than duloxetine (13/20 correct). As such there was no evidence that participants in the duloxetine group were unblinded by any (unreported) side effects. Perceived drug-group membership did not affect emotion recognition.

### Effect of duloxetine on emotion recognition

Mixed-model ANOVA of emotion recognition accuracy scores provides evidence that duloxetine reduced the accurate recognition of sadness compared to placebo, reflected in the drug × time interaction (*F* (1,36) = 4.40, *p* = 0.043, np^2^ = 0.109, 95% confidence interval (CI) (0.003–0.193)), with little evidence of main effects of time (*F* (1,36) = 0.52, *p* = 0.48) or drug (*F* (1, 36) = 0.41, *p* = 0.53). Recognition of sadness improved in the placebo group, *t*(18) = 2.16, *p* = 0.044, and reduced slightly in the duloxetine group, *t*(18) = 0.91, *p* = 0.31. There was no clear evidence of drug group differences at either baseline or follow-up (*t*(36)s < 1.91, *p*s > 0.08). Descriptive statistics are presented in Table 2.

**Table 2.** Emotion recognition in duloxetine and placebo groups on day 0 (baseline) and day 14 (post-drug).

	Duloxetine				Placebo			
	(*n* = 19)				(*n* = 19)			
	Day 0		Day 14		Day 0		Day 14	
*Hits*								
Anger	8.42	(1.90)	8.58	(1.50)	8.63	(1.80)	8.47	(1.35)
Disgust	9.58	(2.37)	9.58	(3.52)	8.74	(2.99)	9.32	(2.20)
Fear	4.95	(2.80)	6.10	(4.24)	4.26	(3.03)	5.37	(3.48)
Happy	12.79	(1.72)	12.58	(1.54)	12.42	(1.47)	11.84	(2.14)
Sad	11.53	(1.95)	11.16	(2.32)	10.63	(1.98)	11.63	(2.14)
Surprise	12.00	(1.29)	12.05	(1.51)	11.68	(1.42)	11.63	(2.22)
*False alarms*								
Anger	1.16	(1.38)	1.58	(1.77)	3.26	(2.96)	2.63	(2.41)
Disgust	2.90	(2.47)	3.63	(2.79)	4.95	(3.50)	4.58	(4.14)
Fear	7.05	(4.27)	6.68	(5.59)	6.39	(4.59)	5.58	(5.20)
Happy	4.90	(5.28)	4.10	(5.92)	4.26	(5.10)	4.63	(5.23)
Sad	4.21	(3.01)	4.79	(4.18)	5.00	(5.09)	5.05	(3.79)
Surprise	10.26	(4.81)	9.11	(5.98)	9.58	(4.93)	9.10	(5.07)
*Recognition accuracy*								
Anger	0.55	(0.13)	0.55	(0.10)	0.53	(0.11)	0.53	(0.09)
Disgust	0.60	(0.16)	0.59	(0.23)	0.52	(0.19)	0.56	(0.14)
Fear	0.24	(0.19)	0.31	(0.31)	0.20	(0.21)	0.29	(0.24)
Happy	0.79	(0.10)	0.78	(0.09)	0.77	(0.07)	0.73	(0.11)
Sad	0.71	(0.12)	0.68	(0.14)	0.65	(0.11)	0.71	(0.13)
Surprise	0.66	(0.09)	0.68	(0.09)	0.65	(0.12)	0.65	(0.16)

Values represent mean (standard deviation).

To what extent do these results reflect changes in *sensitivity* to sadness (i.e. the ability to correctly detect subtle expressions of sadness)? Follow-up tests provide evidence that the drug × time effect on sadness recognition was driven by a change in hit rate (i.e. correct identification of sadness; drug × time, *F* (1,36) = 4.99, *p* = 0.032, np^2^ = 0.122, 95% CI (0.126–2.611) rather than false alarms (i.e. mislabelling of sadness); *F* = 0.11, *p* = 0.70. Furthermore, drug × time interactions on accuracy (and hit rate) were evident only at weaker intensities of sadness (i.e. accuracy across the seven expressions in the lower half of the sad-neutral continuum – see Figure 1), *F* (1,36) = 5.54, *p* = 0.024, np^2^ = 0.133, 95% CI (0.024–0.321), reflecting a large effect size. There was no evidence of effects at stronger intensities, *F*s < 0.48, *p*s > 0.49. Consequently group differences in sadness recognition are characterised by changes in the ability to correctly detect subtle expressions of sadness.

There was no evidence that duloxetine altered the accurate recognition of other emotional expressions overall, or at weaker intensities (*F*s < 1.04, *p*s > 0.32, np^2^ < 0.028 for drug × time interactions). Likewise separate analyses of hits and false alarm rates did not provide any evidence that drug groups differed in their identification or mislabelling of any other emotional expressions over time. Full results for hits and false alarms are presented in Table 2.

## Discussion

The two groups differed in sadness recognition over time, and in particular their ability to detect subtle expressions of sadness. There was no evidence that the duloxetine and placebo groups differed in their accurate recognition, detection or mislabelling of other emotions. Our findings extend evidence that the recognition of negative expressions is reduced following sub-chronic administration of other antidepressants, including SSRIs (Harmer et al., 2004; Murphy et al., 2009), NRIs (Harmer et al., 2009) and the novel antidepressant agomelatine (Harmer et al., 2011); and that a single 60 mg dose of duloxetine can increase the recognition of happy expressions (Harmer et al., 2008). A recent review of the effect of antidepressants on emotion recognition in healthy volunteers suggests that while single doses of antidepressants appear to increase the recognition of positive expressions, sub-chronic (i.e. seven-day) administration appears to exclusively reduce recognition of negative emotion (see Table 1, Pringle et al., 2013). While the temporal changes in emotion recognition following initial to sub-chronic antidepressant administration remain unclear, behavioural findings to date fit with evidence that clinical response to antidepressants tends to be characterised by an initial increase in positive affect, followed by the reduction in negative mood (Geschwind et al., 2011).

In previous studies, SSRI/NRIs have been reliably found to selectively reduce the recognition of fearful faces (e.g. Harmer et al., 2004). In our study fear recognition was unaffected by the SNRI duloxetine, but fear recognition did increase (across all participants) from day 0 to day 14. Fear is one of the more difficult emotions to discriminate in this type of task (see Table 2), and it is possible that our participants’ improved recognition of fear constitutes a practice effect. In addition, participants’ increased recognition of fear on day 14 could result from a general increase in their anxiety (see Table 1), perhaps reflecting their anticipation of the carbon-dioxide challenge that was to be completed later that day. These factors, together with our use of a different face stimulus set, may explain why we did not observe an effect of duloxetine on fear recognition.

We used a novel face recognition task and stimulus set in a repeated-measures design to directly compare group differences in emotion recognition accuracy at baseline and post-drug. To our knowledge previous studies of antidepressant drug effects on emotional face recognition have compared groups post-drug, but not at baseline (see Pringle et al., 2013 for review). These studies assume that participant randomisation procedures adequately control the risk that post-drug group differences reflect (unmeasured) pre-treatment/baseline group differences in performance. We did not find clear evidence of group differences in emotion recognition at baseline or follow-up; rather drug-group differences in sadness recognition were characterised by greater change in the placebo compared to the duloxetine group. The reasons for this are unclear, and further research is required to quantify changes in emotion recognition in those taking duloxetine.

Though repeated-measures designs allow us to compare drug effects over time within and between groups, they do require tests and measures that are sensitive across repeated testing sessions and resistant to practice effects and habituation to test stimuli. We found a large drug × time effect on the ability to *correctly detect* subtle expressions of sadness, but a more modest effect across sad expressions in general. These results suggest that drug-group effects reflect genuine changes in recognition accuracy/sensitivity rather than non-systematic fluctuations in participant responding (e.g. response criterion). However, our analysis method applied multiple statistical tests, and some of our findings fell short of conservative Bonferroni-corrected *p* thresholds. Accordingly, and despite the limitations of using *p* values to assess ‘significance’ (see Cumming, 2014), our preliminary findings require replication. We recommend future longitudinal studies increase power by recruiting larger samples, using a restricted range of emotional intensities, and where possible avoid over-exposing participants to high-intensity expressions that might confound measures of subtle biases in emotion recognition. Drug administration studies should also monitor compliance, and where possible measure drug levels from blood to extend self-report measures and returned-tablet counts used in our study. This will be particularly important when studying the neurocognitive effects of drugs over longer periods, i.e. beyond 14 days and throughout periods when these drugs typically start to achieve therapeutic effects in clinical populations.

How might the effect of antidepressants on the recognition of sadness alleviate depression? We recently showed, in a meta-analytic review, that while major depression is associated with a general deficit in emotion recognition, the recognition of sadness is uniquely preserved. This suggests that in relative terms individuals with major depression may be more sensitive to sadness than other emotions (Dalili et al., 2014). Research in patients with depression suggests that antidepressants can normalise biases in emotional face processing (Harmer et al., 2009). Likewise, administration of the SSRI fluoxetine (20 mg/day) for eight weeks can reduce neural responses to sad faces (Fu et al., 2004), and increase neural responses to happy faces (Fu et al., 2007) in depressed patients. Although the effect of duloxetine on emotion recognition has not been examined in clinical populations, initial evidence in clinical depression suggests that duloxetine treatment can improve social functioning (Oakes et al., 2012), cognitive performance, such as verbal learning and memory (Raskin et al., 2007), attention (Herrera-Guzmán et al., 2010), and episodic memory (Herrera-Guzmán et al., 2009), and lower neural responses to painful stimuli (López-Solà et al., 2010). The neural effects of duloxetine administration have been further clarified in healthy volunteers. Sub-chronic administration of duloxetine (60 mg/day for 14 days) can reduce amygdala activation to negative expressions (van Marle et al., 2011), increase activity in ventral striatal reward networks (Ossewaarde et al., 2011) and reduce the neural correlates of emotional memory formation in a sad mood induction procedure (Tendolkar et al., 2011).

Results to date suggest that duloxetine can produce adaptive changes in behavioural and neural mechanisms that characterise low mood, consistent with growing evidence that antidepressants can modulate emotion recognition and social functioning in clinical depression. However, the effects of antidepressants on emotion recognition in clinical anxiety are not known. Evidence suggests that biases in emotion recognition might differ across anxiety disorders (Garner et al., 2009), and that biases that characterise anxiety might differ from those that characterise major depression (Demenescu et al., 2010). If so, different pharmacological agents might be used to selectively target the biases that characterise different disorders. This approach would be consistent with the broader goals of stratified and trans-diagnostic medicine, in which treatments are selected to target mechanisms (e.g. neuropsychological bias), rather than a combination of symptoms. To this end, studies in healthy volunteers and clinical populations should better understand the strength of associations between changes in neuropharmacology, neural activity in emotion networks and behaviour (see Ossewaarde et al., 2011), and examine relationships with dose-response and drug-levels. In addition, designs that directly compare the effects of different drug classes (Harmer et al., 2004) will help delineate the role of different neurotransmitters (e.g. serotonin, noradrenalin, melatonin) and receptor subtypes in adapting neuropsychological mechanisms (e.g. biases in attention, interpretation, memory – see Pringle et al., 2013 for discussion). This approach might also promote the pharmacological augmentation of cognitive-behavioural psychological interventions that directly target neuropsychological biases, such as emotion recognition, to improve clinical outcomes (Adams et al., 2013; Browning et al., 2011; Penton-Voak et al., 2012). Future studies might also examine whether the return of maladaptive biases in emotion processing precedes relapse in those who have responded to treatment. If so, interventions that target these mechanisms have potential to be employed in relapse prevention approaches.

To conclude, our study is the first to examine the behavioural effects of sub-chronic duloxetine administration on emotion recognition. Evidence that sub-chronic administration of duloxetine may reduce recognition of sadness is consistent with cognitive neuropsychological models of antidepressant drug action (Harmer and Cowen, 2013) and highlights a mechanism through which SNRIs might achieve their therapeutic effects.
